# Central Pontine Myelinolysis Leading to an Intensive Care Unit Admission

**DOI:** 10.7759/cureus.106210

**Published:** 2026-03-31

**Authors:** Miguel Santos, Ana Cunha, Ana Alburquerque, Eugeniu Gisca, Fernando Henriques, Odete Gomes

**Affiliations:** 1 Intensive Care Unit, Unidade Local de Saúde da Região de Leiria, Leiria, PRT

**Keywords:** central pontine myelinolysis, hyponatremia, medical intensive care unit (micu), osmotic demyelination syndrome (ods), treatment of hyponatremia

## Abstract

Osmotic demyelination syndrome (ODS) is a rare and serious clinical syndrome that has been associated with rapid correction of severe hyponatremia. ODS can evolve to central pontine myelinolysis (CPM), in which demyelination occurs in the pontine region, and less commonly extrapontine myelinolysis (EPM), involving other brain regions. We present the case of a 52-year-old alcoholic man admitted for community-acquired pneumonia who developed CPM following rapid correction of severe hyponatremia. This case highlights the importance of careful correction of sodium levels, particularly in high-risk patients.

## Introduction

Osmotic demyelination syndrome (ODS) is a rare and serious clinical syndrome that has been associated with rapid correction of severe hyponatremia [1]. ODS can evolve to central pontine myelinolysis (CPM), in which demyelination occurs in the pontine region, and less commonly extrapontine myelinolysis (EPM), involving the white matter of the cerebral hemispheres [1,2]. Adams et al. first described CPM in 1959 [2], and it was later observed that a significant proportion of cases are associated with rapid correction of hyponatremia, although other risk factors have been identified, including chronic alcohol use, malnutrition, and hyperemesis [3-5]. The incidence of ODS is not well established, but it is considered rare, and the increased availability of MRI has contributed to improved recognition of this condition [6]. The severity and duration of hyponatremia, as well as the rate of correction, are important factors influencing the risk of ODS, and current recommendations emphasize cautious correction, particularly in high-risk patients [7]. We present the case of a 52-year-old alcoholic man admitted for community-acquired pneumonia who developed CPM in the context of rapid correction of severe hyponatremia.

## Case presentation

A 52-year-old man with a history of chronic alcohol use presented to the emergency department with a suspected seizure episode. He was found lying on the ground with loss of sphincter control and reported anorexia and sweating in the previous days. On physical examination, he was tachycardic, polypneic, and diaphoretic, while neurological examination was unremarkable at admission.

**Figure 1:**
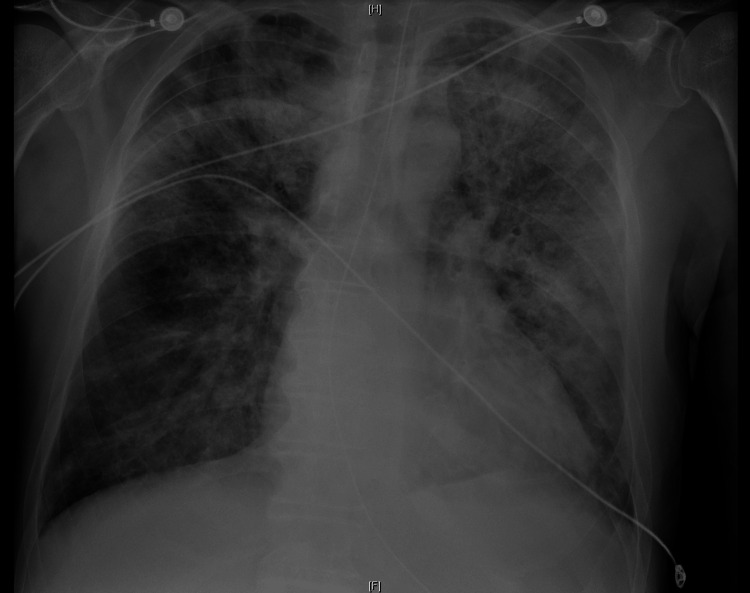
Chest X-ray evidencing bilateral pneumonia

**Figure 2:**
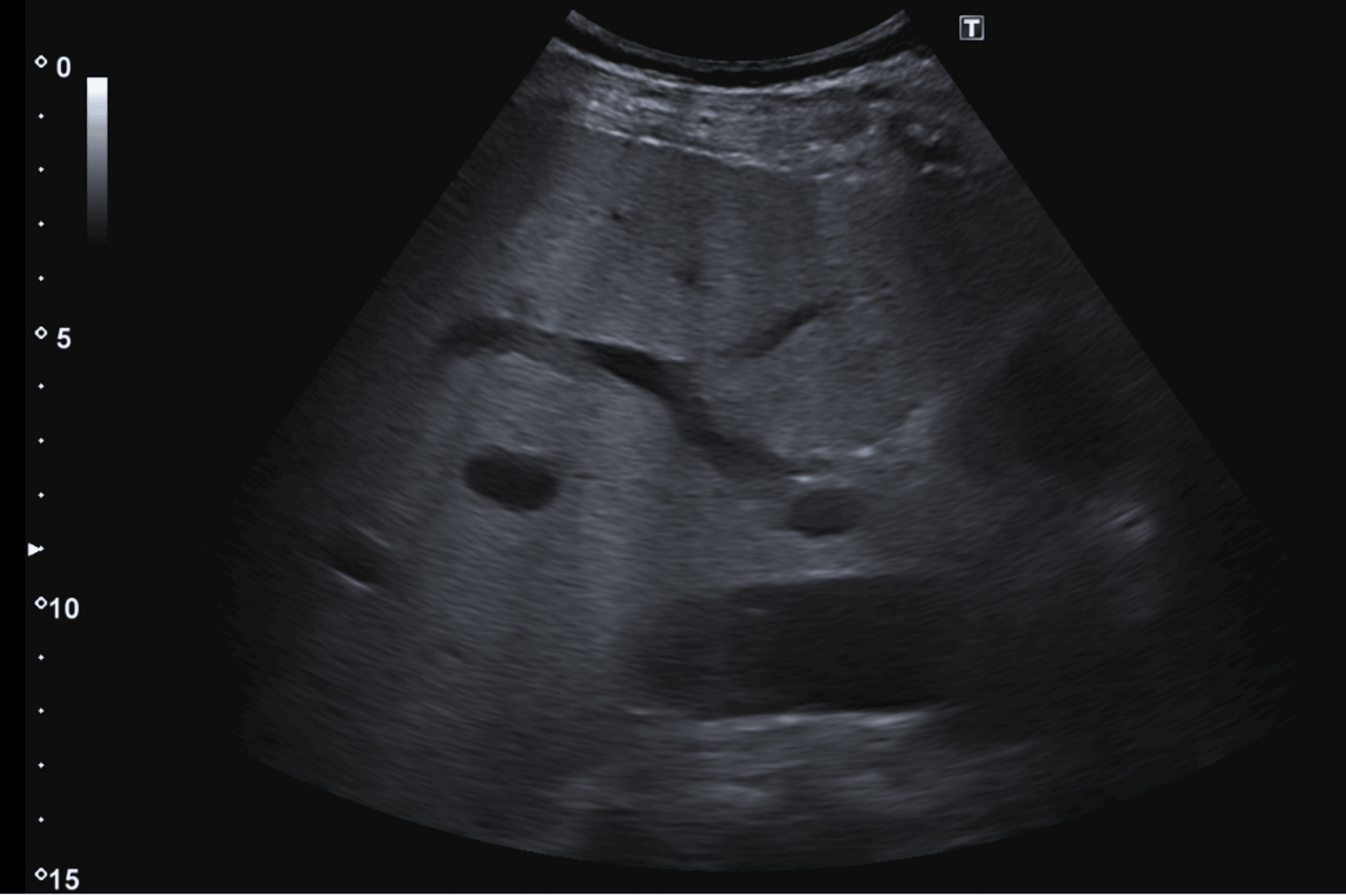
Abdominal ultrasound Abdominal ultrasound report highlights a large homogenous but hyperechoic liver, correlating to chronic liver disease.

Laboratory results revealed severe hyponatremia (serum sodium 104 mmol/L), thrombocytopenia, prolonged prothrombin time and INR, hyperbilirubinemia, elevated alanine aminotransferase (ALT) and aspartate aminotransferase (AST), and elevated C-reactive protein (Table 1). Chest X-ray showed bilateral pneumonia (Figure 1), and abdominal ultrasound demonstrated findings suggestive of chronic liver disease (Figure 2), while a head CT scan was normal.

**Table 1:** Laboratory results at admission ALT: Alanine aminotransferase; AST: Aspartate aminotransferase

Parameters	Patient Values	Reference Range
Platelet count	82,000 U/L	150,000-50,000 U/L
Prothrombine time	21.3s	9.40-12.50s
INR	1.91 ratio	0.50-1.2 ratio
Serum sodium concentration	104 mmol/L	136-145 mmol/L
Serum potassium concentration	5.6mmol/L	3.5-5.0 mmol/L
Total bilirubin	167.7 μmol/L	5.0-21.0 μmol/L
ALT	476 U/L	3-45 U/L
AST	1,150 U/L	15-50 U/L
C-reactive protein	40 mg/dL	<5.0 mg/dL

The patient was started on intravenous antibiotics and isotonic saline. The use of isotonic saline was guided by the overall clinical context, including suspected infection and subsequent hypotension requiring fluid resuscitation. During the first 48 hours of hospitalization, a rapid increase in serum sodium levels was observed, rising from 104 mmol/L at admission to 131 mmol/L. At the time of ICU admission, serum sodium had further increased to 152 mmol/L, exceeding recommended correction rates for high-risk patients.

On day eight, the patient developed fever and significant neurological deterioration, with a Glasgow Coma Scale of 8, requiring admission to the ICU and mechanical ventilation. Laboratory tests at ICU admission showed leukocytosis and elevated inflammatory markers (Table 2), while cerebrospinal fluid analysis was unremarkable and repeat CT scan was normal.

**Table 2:** Blood laboratory results at admission to ICU

Parameters	Patient Values	Reference Range
Leukocyte count	13.300 U/L	4.0-10.0 U/L
Neutrophil count	10.600 U/L	1.8-8.0 U/L
C-reactive protein	64.8 mg/dL	<5.0 mg/dL
Serum sodium concentration	152 mmol/L	136-145 mmol/L

**Figure 3:**
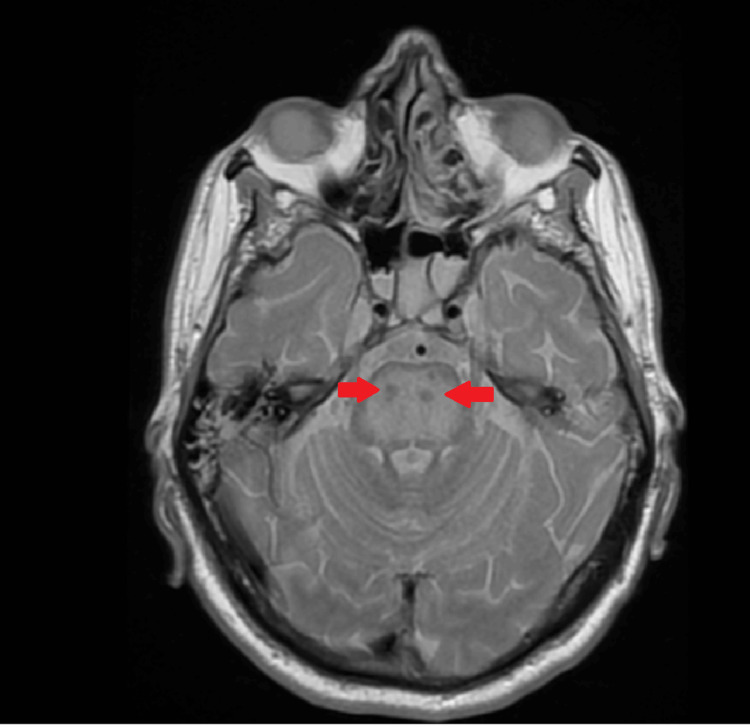
MRI T1 axial image demonstrating low signal in the pons (red arrows)

**Figure 4:**
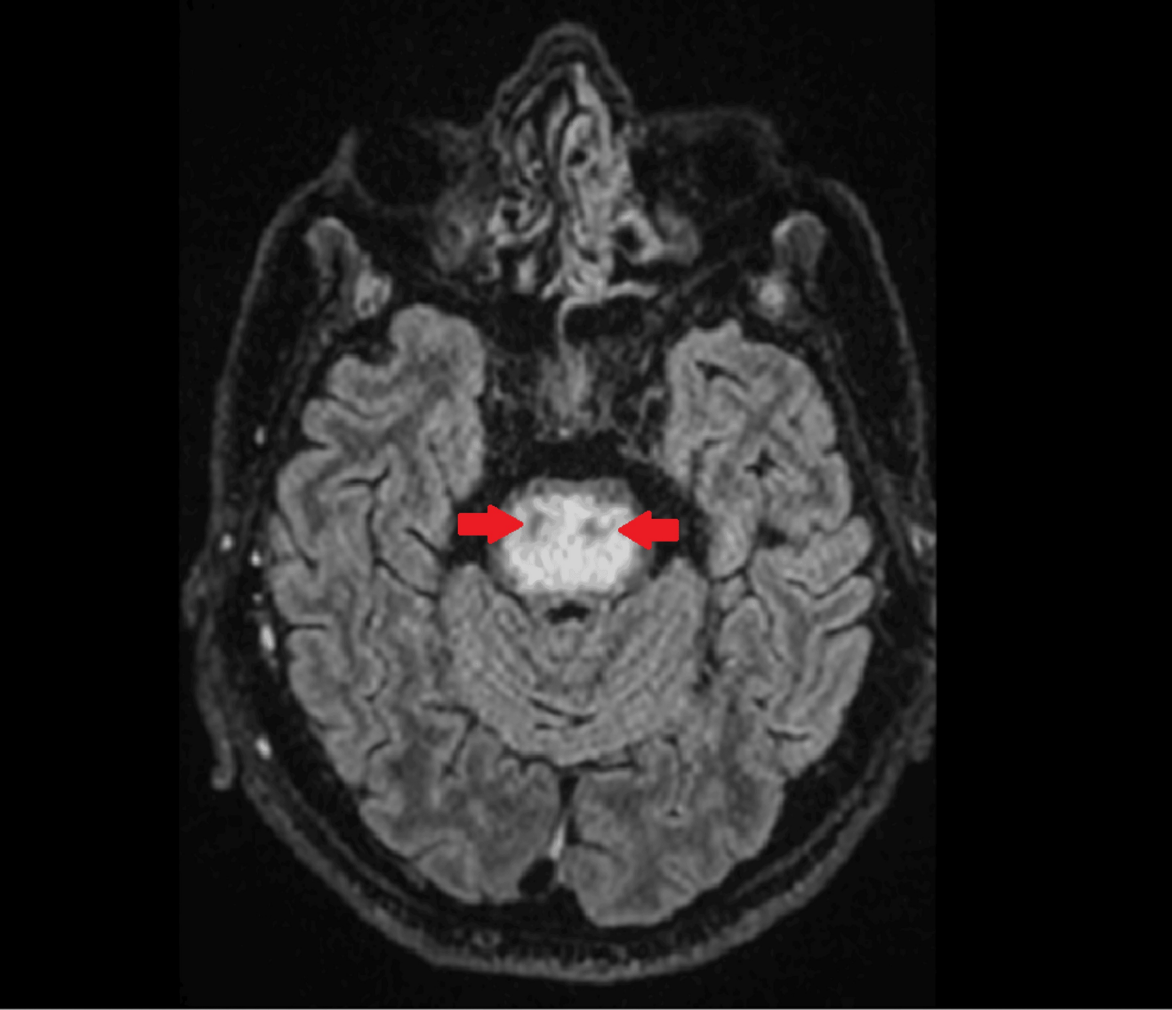
MRI FLAIR axial image demonstrating low signal in the pons (piglet sign) (red arrows) FLAIR: Fluid-attenuated inversion recovery

At this time, 0.45% sodium chloride solution was used to lower serum sodium, and thiamine was administered given the history of chronic alcohol use. MRI revealed characteristic findings, with low signal intensity on T1 and high signal intensity on T2/fluid-attenuated inversion recovery (FLAIR) in the pons (Figures 3, 4), consistent with CPM.

During ICU stay, complications included ventilator-associated pneumonia and iatrogenic pneumothorax, contributing to prolonged hospitalization. Supportive management included mechanical ventilation, electrolyte monitoring, and nutritional support. After withdrawal of sedation, the patient exhibited tetraparesis with preserved eye movements, and progressive neurological improvement was observed with rehabilitation. At discharge, the patient was conscious, with residual dysphagia and moderate motor weakness, and was transferred to a rehabilitation unit.

## Discussion

Our patient had multiple risk factors associated with ODS, including chronic alcohol use, probable chronic liver disease, and severe hyponatremia at presentation. A rapid increase in serum sodium levels was observed during hospitalization, exceeding recommended correction thresholds [7], and rapid correction of hyponatremia has been associated with the development of ODS, particularly in high-risk patients [3-5].

Neurological deterioration in critically ill patients may have multiple causes, including metabolic encephalopathy or infection. In this case, cerebrospinal fluid analysis was unremarkable, and brain CT scans did not show acute abnormalities. The diagnosis of CPM was supported by characteristic MRI findings in the appropriate clinical context, and MRI remains the main diagnostic modality for ODS, typically demonstrating characteristic pontine signal abnormalities [8].

Although the prognosis of CPM is often considered poor, recovery is possible, as illustrated in this case [9]. This case highlights the importance of careful monitoring and controlled correction of hyponatremia, particularly in patients with multiple risk factors, and reinforces that prevention remains the most effective strategy.

## Conclusions

This case highlights the importance of careful clinical evaluation and close monitoring of sodium correction in hospitalized patients, particularly those at high risk. ODS remains a rare but serious neurological condition that has been associated with rapid correction of hyponatremia. Early recognition and strict adherence to recommended correction rates are essential to minimize the risk of neurological complications and improve patient outcomes.
